# High risk human papillomavirus prevalence and genotype distribution among women infected with HIV in Manaus, Amazonas

**DOI:** 10.1186/s12985-018-0942-6

**Published:** 2018-02-17

**Authors:** Monique Figueiredo Teixeira, Meritxell Sabidó, André Luiz Leturiondo, Cynthia de Oliveira Ferreira, Kátia Luz Torres, Adele Schwartz Benzaken

**Affiliations:** 1 Universidade Federal do Amazonas (UFAM), 6200, Coroado I, General Rodrigo Octávio Ave, Manaus, Amazon 69080-900 Brazil; 2 TransLab. Departament de Ciències Mèdiques, Facultat de Medicina, Girona, Catalunya Spain; 3 CIBER de Epidemiología y Salud Pública (CIBERESP), Madrid, Spain; 4 Fundação Alfredo da Matta (FUAM), Manaus, Amazonas Brazil; 5 Fundação Centro de Controle de Oncologia do Amazonas (FCecon), Manaus, Amazonas Brazil; 6 Fundação de Medicina Tropical Dr. Heitor Vieira Dourado (FMT-HVD), Manaus, Amazonas Brazil; 7 Departamento de IST, Aids, e Hepatites Virais, Ministério da Saúde, Brasília, DF Brazil

**Keywords:** Human papillomavirus, Human immunodeficiency virus, Prevalence, Cytology, Polymerase chain reaction

## Abstract

**Background:**

Human immunodeficiency virus (HIV)-positive women have a high prevalence of human papillomavirus (HPV), and are infected with a broader range of HPV types than HIV-negative women. We aimed to determine the prevalence of cervical cytologic abnormalities, high-risk (HR)-HPV prevalence, type distribution according to the severity of cervical lesions and CD4 cell count and identify factors associated with HR-HPV infection among women living with HIV in Manaus, Amazonas.

**Methods:**

We enrolled 325 women living with HIV that attended an infectious diseases referral hospital. Each woman underwent a gynecological exam, cervical cytology, HR-HPV detection by Polymerase chain Reaction (PCR) using the BD Onclarity™ HPV Assay, colposcopy and biopsy, when necessary. We assessed the associations between potential risk factors and HR-HPV infection.

**Results:**

Overall, 299 (92.0%) women had a PCR result. The prevalence of HR-HPV- infection was 31.1%. The most prevalent HR-HPV types were: 56/59/66 (32.2%), 35/39/68 (28.0%), 52 (21.5%), 16 (19.4%), and 45 (12.9%). Among the women with HR-HPV infection (*n* = 93), 43.0% had multiple infections. Women with HPV infection showed higher prevalence of cervical abnormalities than that HPV-negative (LSIL: 22.6% vs. 1.5%; HSIL: 10.8% vs. 0.0%). The prevalence of HR-HPV among women with cytological abnormalities was 87.5% for LSIL and 100.0% for HSIL. Women with CD4 < 200 cell/mm^3^ showed the highest HR-HPV prevalence (59.3%) although this trend was not statistically significant (*p*-value = 0.62). The mean CD4 cell count decreased with increasing severity of cervical lesions (*p*-value = 0.001). The multivariable analysis showed that increasing age was associated with a decreased risk of HR-HPV infection with an adjusted prevalence odds ratio of 0.9 (95.0% CI: 0.9–1.0, *p*-value: 0.03) for each additional year. The only factor statistically significant associated with HR-HPV infection was CD4 cell count.

**Conclusions:**

HR-HPV and abnormal cytology prevalence are high among women in the Amazonas. The low CD4 cell count was an important determinant of HPV infection and abnormal cytological findings. HPV quadrivalent vaccination used in Brazil might not offer protection for an important fraction of HPV-related disease burden in women living with HIV. This is partly explained by the high presence of non targeted vaccine HR-HPVs, such as the HPV genotype groups 56/59/66, 35/39/68 and individually HPV-52 and HPV-45, some of which contribute to high-grade lesion.

**Electronic supplementary material:**

The online version of this article (10.1186/s12985-018-0942-6) contains supplementary material, which is available to authorized users.

## Background

Infection with human papilloma virus (HPV) is the main cause of cervical cancer [1]. In Brazil, it is estimated that approximately 10.7% of women in the general population with normal cytology have cervical HPV infection [2]. In the state of Amazonas, HPV has been shown to be the most prevalent sexually transmitted infection (STI) in the population [3].

Young women are the most affected by HPV and by multiple infections. The prevalence tends to decrease with increasing age [4]. A high viral load and the persistence of oncogenic HPV types are progression factors for precancerous lesions and cervical cancer [5]. Additional factors might influence the development of precursor lesions or cancer, such as those related to immunity, genetics and sexual behaviour. In women over 30 years, HPV infection tends to be more persistent than in younger women [6].

Women living with HIV have a higher prevalence of HPV infection with high-risk oncogenic (HR-HPV) multiple infections. Immunosuppression resulting from HIV increases the risk of developing squamous intraepithelial lesions when compared with the general population [7–10]. Patients more severely immunocompromised as a result of HIV infection might have a higher incidence and persistence of lesions caused by HPV [8].

It is also possible that adherence to highly active antiretroviral therapy (HAART) is associated with decreased development of precursor lesions of cervical cancer and improved clearance of HPV infection, increasing survival of women living with HIV with a consequent decrease in cases of cervical cancer [11].

In Amazonas, Brazil, there are few data on the epidemiology of HPV and related cancers and the impact of HIV on these conditions. The objective of this study was to estimate the prevalence of cervical HPV infection and the frequency of genotypes, according to the severity of cervical lesions and CD4 cell counts and identify factors associated with HPV infection in women living with HIV/AIDS that attended a reference hospital for HIV/AIDS in Manaus, Amazonas.

## Methods

### Study design

A cross-sectional study was conducted for HR-HPV screening in women living with HIV/AIDS that attended an outpatient HIV reference service within a tertiary care hospital (FMT-HVD). This teaching hospital attends most of the HIV/AIDS cases in the Amazon state (95.0%). This reference hospital is the unique ART provider in the Amazonas state. The study was performed from May 2014 to February 2015.

### Study participants

Women who had a confirmed HIV diagnosis and consecutively sought a gynecological visit to perform routine cytology in the HIV outpatient service of the FMT-HVD hospital were eligible for the study. Women were included if older than 18 years of age, agreed to sign the consent form, were not pregnant and did not have a contraindication for Pap smear examination (i.e., current use of vaginal ovules, menstruation, vaginal clean-up during the last 24 h. In case of contraindication they were rescheduled after conditions were resolved. Hysterectomized women were excluded.

In this study, we aimed to include 323 women living with HIV, based on a prevalence of HPV infection in women living with HIV/AIDS of 65.2%, [12] with 80.0% power and assuming a 5.0% level of significance.

### Data and sample collection

After signing the consent form, a nurse interviewed women using a structured questionnaire. The questionnaire included items on sociodemographic, clinical, behavioural, reproductive health and HIV history, including current antiretroviral therapy (ART) use and previous change in ART regimen, and current STI signs and symptoms. Data on CD4 cell counts (cells/mm^3^) and detectable viral load (copies/mL), and nadir CD4 cell count (cells/mm^3^) were obtained from electronical medical records of the hospital. However, when the last determination had been undertaken more than three months before enrolment, a blood sample was collected.

The participants underwent a gynecological evaluation and two samples of cervical cells were collected. The first sample was taken for conventional cytology using a long Ayres’s spatula for subsequent processing at the hospital. The second sample was collected with a cervical brush (*Rovers Cervex-Brush Combi*®, Rovers Medical Devices B.V. Oss, the Netherlands) and introduced into *SUREPATH*® *Preservative Fluid* (TriPath Imaging, Burlington, NC). Samples were transported on the same day at room temperature to the Fundação Centro de Controle de Oncologia (FCECON) laboratory, where 1 mL of each sample was stored at − 80 °C until they were shipped for HPV determination.

### Cervical cytology

Cervical cytology samples were processed at the Department of Pathology of the FMT-HVD. The smears were stained with the Papanicolaou stains and the 2001 Bethesda system was used for classification of cytology results [13]. Cytology examination was carried out under blinded conditions and independently of HPV detection results in PCR by two cytopathologists. A third cytopathologist evaluated discordant results, and if discordancy persisted, agreement was reached between the three.

### HPV detection and typing

Samples for HR-HPV determination were shipped to the Instituto de Câncer do Estado do São Paulo (ICESP). They were processed using the BD Onclarity™ HPV Assay (BD Diagnostics, Sparks, MD), which can detect 14 HR-HPV genotypes by simultaneous identification of the HR types 16, 18, 31, 45, 51, 52, and due to the limits of this test, other HR genotypes reported by genotype group (33/58; 56/59/66; 35/39/68). The BD Onclarity™ HPV Assay has shown good performance when compared with Hybrid Capture 2, with specificity ranging from 50.3 to 95.2% and sensitivity from 95.2 to 98.0% [14, 15]. Molecular testing was performed using the automated BD Viper™ LT System (BD Diagnostics, Sparks, MD). HR-HPV detection was carried out under blinded conditions with regard to subjects’ characteristics and cytology results.

### Data analysis

Data were analyzed using Stata 10.0 (StataCorp LP, College Station, TX). Data were described using percentages and medians with interquartile ranges (IQR), as appropriate. Prevalence and 95.0% confidence interval (CI) were calculated. The results were categorized according to CD4 cell counts (< 200; 200–499; ≥500 cells/mm^3^). Comparisons between HR-HPV infected and non-infected women were formally carried out using for categorical variables the X^2^ test, and for continuous variables, the student-t test or the Fisher exact test (when expected frequencies were less than 5), or the U-Mann Whitney test (for non-parametric variables). To ascertain associations between potential risk factors and HR-HPV infection, prevalence odds ratios (pOR) were calculated with their corresponding 95.0% CI. For the multivariate analysis, pOR were calculated by multiple logistic regression modelling that included covariates for potential confounders, and for factors that were statistically significant (*p* < 0.1) at univariate analysis. All tests were two-tailed and the *p*-value less than 0.05 was considered statistically significant.

The agreement for the blinded and independently cytology reading was measured through the percentage of overall agreement, the percentage of positive agreement, percentage of negative agreement, and the prevalence-adjusted bias-adjusted (PABA)-kappa coefficient, by lesion severity.

### Ethics

The study was approved by the Ethical Institutional Review Board of FMT-HVD (number: 466/2012). Patients gave their signed consent to participate. All women were informed about conventional cytology and HR-HPV detection results. Colposcopy and biopsy were performed following recommendations of the Brazilian Ministry of Health [16]. The study is reported following the STROBE statement and using its checklist for cross-sectional studies [17].

## Results

### Study population description

A total of 331 women were pre-screened and all agreed to participate in the study. Among these, six were excluded because no HIV positive result could be documented in the medical record. Thus, the total number of participants available for analysis was 325. Their median age was 40.7 years (IQR: 33.1–46.2). A total of 299 women living with HIV had a valid PCR result and 324 a valid result in conventional cytology. The median CD4 cell count with IQR among ART users and ART-naïve when HIV was diagnosed was 321 (173–487) and 620 (422–739). The median (IQR) CD4+ cell count among ART users and patients not on ART was 257 (133–283) and 197.5 (88–314.5), respectively.

Table 1 shows the socio-demographic, risk behaviour, reproductive health and HIV history, and current sexually transmitted infections signs and symptoms. Women living with HIV with an HR-HPV-positive result were younger than those with HR-HPV-negative results (median in years: 38.8 vs. 41.1, *p*-value= 0.3). A higher proportion of those with an HR-HPV-positive result had not performed previously cervical cytologies than those with an HR-HPV-negative result (75.0% vs. 25.0%, *p*-value= 0.02), and most frequently had CD4 cells with counts< 200 cells/mm^3^ than women with HP-HPV-negative result (40.7% vs. 59.3, *p*-value=0.001). In general, in each age group, women with a CD4 cell count < 200 cells/mm^3^ had a higher HR-HPV prevalence than women with higher CD4 cell counts, although 95.0% CI was large, suggesting a small number In the CD4 cell count < 200 cells/mm^3^ category.

**Table 1:** Description of population characteristics and results of bivariable and multivariable analysis for risk factors related to HR-HPV infection among women living with HIV in Manaus, Amazonas

Variables	HR-HPV negative *n* = 206 N (%) Median (IQR)	HR-HPV positive *n* = 93 N (%) Median (IQR)	Crude pOR (95.0% CI)	*p*-value	Adjusted pOR (95.0% CI)	*p*-value
Sociodemographic						
Age in years (*N* = 299)	41.1 (45.9–33.5)	38.8 (31.2–44.4)	0.9 (0.9–1.0)	0.03	0.9 (0.9–1.0)	0.03
≤ 34	56 (27.2)	35 (37.6)	1			
35–39	30 (14.6)	18 (19.3)	1.0 (0.5–2.0)	0.91	…	…
40–44	55 (26.7)	18 (19.4)	0.5 (0.3–1.0)	0.27	…	…
≥ 45	65 (31.5)	22 (23.7)	0.5 (0.3–1.0)	0.06	…	…
Civil status (*N* = 299)						
Married/cohabitating	133 (64.6)	50 (53.8)	1			
Single/not cohabitating	73 (35.4)	43 (46.2)	1.5 (0.9–2.5)	0.08	…	…
Level of education (*N* = 299)						
< Primary school	42 (20.4)	25 (26.8)	1			
At least primary school	164 (79.6)	68 (73.2)	1.4 (0.8–2.5)	0.21	…	…
Currently working (*N* = 299)						
Yes	83 (40.3)	31 (33.3)	1			
No	123 (59.7)	62 (66.7)	1.3 (0.8–2.2)	0.25	…	…
Sexual behaviour and other risk behaviour						
Current smokers (*N* = 299)						
No	191 (92.7)	88 (94.6)	1			
Yes	15 (7.3)	5 (5.4)	0.7 (0.2–2.0)	0.54	…	…
Age at first sex (years) (*N* = 297)						
≤ 15	108 (52.4)	44 (48.4)	0.8 (0.5–1.3)	0.52	…	…
> 15	98 (47.6)	47 (51.6)	1			
Sexual partners in life to date (*N* = 297)						
< 4	60 (29.3)	28 (30.4)	1			
4 to 7	65 (31.7)	32 (34.8)	1.0 (0.5–1.9)	0.87	…	…
≥ 8	80 (39.0)	32 (34.6)	0.8 (0.4–1.5)	0.62	…	…
Regular partner currently (*N* = 299)						
Yes	148 (71.8)	62 (66.7)	1			
No	58 (28.2)	31 (33.3)	1.2 (0.7–2.1)	0.37	…	…
Condom use at last sex with regular partner (*N* = 210)						
Yes	107 (72.3)	45 (72.6)	1			
No	41 (27.7)	17 (27.4)	0.9 (0.5–1.9)	0.97	…	…
Occasional sex partner currently (N = 299)						
No	184 (89.3)	82 (88.2)	1			
Yes	22 (10.7)	11 (11.8)	1.1 (0.5–2.4)	0.78	…	…
Condom use at last sex with occasional partner (*N* = 33)						
Yes	16 (72.7)	8 (72.7)	1			
No	6 (27.3)	3 (27.3)	1.0 (0.2–5.0)	1.00	…	…
Reproductive and sexual health						
Current oral contraceptive use (*N* = 299)						
Yes	202 (98.1)	90 (96.7)	1			
No	4 (1.9)	3 (3.3)	1.6 (0.3–7.6)	0.50	…	…
Previous cervical cytology (*N* = 299)						
Yes	204 (99.0)	87 (93.6)	1			
No	2 (1.0)	6 (6.4)	7.0 (1.3–35.5)	**0.02**	…	…
Ever had an STI (*N* = 299)						
No	146 (70.9)	65 (69.9)	1			
Yes	60 (29.1)	28 (30.1)	1.0 (0.6–1.7)	0.86	…	…
Parity (N = 299)						
Nulliparous	11 (5.3)	10 (10.8)	1			
1 to 3	97 (47.0)	39 (41.9)	0.4 (0.1–1.1)	0.09	…	…
≥ 4	98 (47.7)	44 (47.3)	0.4 (0.2–1.2)	0.14	…	…
Ever had an abortion (*N* = 278)						
No	90 (46.2)	44 (53.0)	1			
Yes	105 (53.8)	39 (47.0)	0.7 (0.4–1.2)	0.30	…	…
HIV history						
Time since HIV diagnosis in years (N = 298)	6 (3–10)	5 (2–10)	0.9 (0.9–1.0)	0.53	…	…
CD4 cell count nadir^b^ (*N* = 294)	195 (83–317)	221 (86–357)	1.0 (0.9–1.0)	0.90	…	…
CD4 cells/mm^3a^ (N = 298)	348 (197–550)	322 (162–491)	0.9 (0.9–1.0)	0.30		
≥500	115 (56.1)	36 (38.7)	1		1	
200–499	79 (38.5)	41 (44.1)	1.6 (0.9–2.8)	0.06	1.6 (0.9–2.8)	0.06
< 200	11 (5.4)	16 (17.2)	4.7 (2.0–11.3)	**< 0.001**	4.7 (2.0–11.3)	**< 0.001**
Detectable viral load (copies/mL)^a^ (*N* = 298**)**	6049 (390–32,000)	11,112.5 (1070–43,258.5)	1.0 (0.9–1.0)	0.60		
Current ART (*N* = 299**)**						
Yes	185 (89.8)	78 (83.9)	1			
No	21 (10.2)	15 (16.1)	1.6 (0.8–3.4)	0.14	…	…
Previous change in ART regimen (*N* = 270)						
No	119 (62.9)	44 (54.3)	1			
Yes	70 (37.1)	37 (45.7)	1.4 (0.8–2.4)	0.18	…	…

*ART* Antiretroviral therapy, *CI* Confidence interval, *IQR* Interquartile range, *pOR* Prevalence odds ratio, *SD* Standard deviation, *STI* Sexually transmitted infection, *p*-value < 0.05: statistically significant

^a^Most recent blood test collected within 3 months before enrolment

^b^It is the lowest CD4 cell count of the patient

### HR-HPV prevalence, associated factors, and genotype distribution by age and CD4 cell count category

**Fig. 1:**
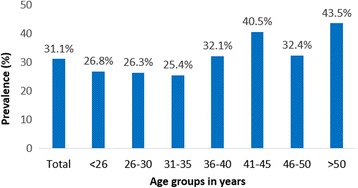
Prevalence of HPV infection according to age among 299 women living with HIV in Manaus, Amazonas

The results of the PCR screening showed that 93 out of 299 women were infected with HR-HPV, resulting in a prevalence of 31.1% (95.0% CI: 25.8–36.4). The distribution of age specific HR-HPV prevalence ranged from 25.4% in the age group 31–35 years to 43.5% in those aged > 50 years. The proportion seemed to increase from 36 years onwards, peaking at older women (Fig. 1) although this pattern was not statistically significant (*p*-value = 0.07). HR-HPV prevalence by CD4 cell group was 23.8% in those with CD4 > 500 cell/mm^3^, 34.2% in 200–499 cell/mm3 and 59.3% in < 200 cell/mm^3^. The proportion increased with decreasing CD4 cell count, although this pattern was not statistically significant (*p*-value = 0.62).

**Fig. 2:**
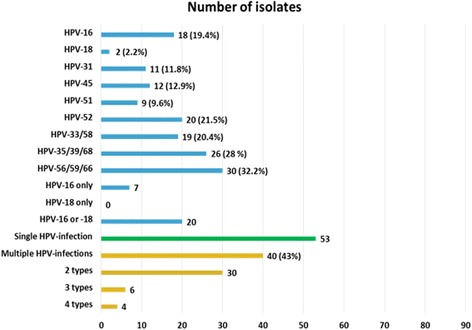
HPV genotypes distribution among 93 HIV-HPV positive women in Manaus, Amazonas

The most prevalent HR-HPV types among 93 HR-HPV-positive and HIV positive women were HPV pool-56/59/66 (32.3%), HPV-35/39/68 (28.0%) and isolated HPV-52 (21.5%), HPV-58 (20.4%), HPV-16 (19.4%), HPV-45 (12.9%), HPV-31 (11.8%), and HPV-18 (2.2%) (Fig. 2). Multiple infections (range: 2–4 types) were identified in 40 of 93 women (43.0%). The prevalence of women with multiple HR-HPV infections was 13.4% (40/299) (95.0% CI: 9.5%–17.3). Among cases of multiple infection (*N* = 40), the prevalence of HPV-16 was 27.5% (*n* = 11), of HPV-18 was 5.0% (*n* = 2) and of HPV-52 was 35.0% (*n* = 14). Multiple infections had a higher HR-HPV prevalence in older age groups (41–45 years: 18.9%; 46–50 years: 18.9%; > 50 years: 17.4%) than in younger age groups (< 26 years: 7.3%; 26–30 years: 13.2%; 31–35 years: 11.9%) but no trend with age was found (*p-value* = 0.10).

When compared with HR-HPV-negative women, those with a HR-HPV-positive result were younger, a lower proportion had undertaken a cervical cytology ever, had a lower median CD4 cell count at last determination, and a higher proportion had CD4 cell count < 200 cell/mm^3^ (Table 1). Results from the multivariable analysis showed that increasing age was associated with a decreased risk of HR-HPV infection with an adjusted pOR of 0.9 (95.0% CI: 0.9–1.0, *p*-value= 0.03) for each additional year. The only factor statistically significant associated with HR-HPV infection was CD4 cell count: women with CD4 cell count < 200 cell/mm^3^ had an pOR of having HR-HPV that was 4.7 (95.0% CI: 2.0–11.3, *p*-value < 0.001) times greater than that of women with CD4 cell count ≥500 cells/mm^3^.

### Relationship between HR-HPV infection, conventional cytology results, and CD4 cell counts

Overall, 84.2% (275) of women had a normal cytology, 2.7% (9) ASCUS, 1.7% (5) ASC-H, 8.1% (25) LSIL, 3.4% (10) HSIL, and no cancer cases. The proportion of cytological abnormalities was higher in women with HR-HPV-positive than in women with a negative HR-HPV-result. (Additional file 1: Table S1).

By cytological grade, the prevalence of HR-HPV was 37.5% in ASC-US, 80.0% in ASC-H, 87.5% in LSIL, and 100.0% in HSIL. The prevalence of HR-HPV increased with the degree of cytological abnormalities, ranging from 21.9% among women with normal cytology to 100.0% among women with HSIL (*p*-value for trend < 0.0001). The same pattern was observed by decreasing CD4 cell count groups (Table 2).

**Table 2:** HR-HPV prevalence as determined by PCR, according to the cytological findings and HIV viral load among women living with HIV in Manaus, Amazonas

HR-HPV Prevalence				
Cytology	General	CD4 ≥ 500 cell/mm^3^	CD4 200–499 cell/mm^3^	CD4 < 200 cell/mm^3^
Unsatisfactory	0	0	0	0
Normal	55/251 (21.9)	27/139 (19.4)	20/94 (21.3)	8/17 (47.1)
ASC-US	3/8 (37.5)	1/1 (100)	1/4 (25)	1/3 (33.3)
ASC-H	4/5 (80.0)	2/2 (100)	2/3 (66.7)	0
LSIL	21/24 (87.5)	4/6 (66.7)	13/14 (92.9)	4/4 (100)
HSIL	10/10 (100)	2/2 (100)	5/5 (100)	3/3 (100)

*ASC-H* Atypical Squamous Cells of Undetermined Significance, when it is not possible to disregard high degree lesions; *ASC-US* Atypical Squamous Cells of Undetermined Significance, *LSIL* Low-grade Squamous Intraepithelial Lesions, *HSIL* High-grade Squamous Intraepithelial Lesions, *HR-HPV* High Risk Human Papillomavirus

HPV- 56/59/66 (either 56, 59, 66 or any combination of these three types) was the most prevalent genotype (52.3%) among 21 HR-HPV-positive women with LSIL and the most prevalent one (60.0%) among 10 HR-HPV-positive women with HSIL in a total of 93 women coinfected with HR-HPV and HIV (Table 3). The second most common genotypes for LSIL were HPV-16 (23.8%) and HPV-35/39/68 (23.8%), whereas for HSIL HPV-16 was found in half (50.0%) of these women. None of the women with LSIL and HSIL were infected with HPV-18. The prevalence of HR HPV genotype increased by cytological grade in HPV-16, HPV-31, HPV-45, HPV-33/58, and HPV- single infection and multiple infection (*p*-value for trend <0.001), and in HPV-52 (*p*-value for trend = 0.04).

**Table 3:** Distribution of HR-HPV genotype according to cytology results in 93 HPV-HIV positive women in Manaus, Amazonas

	Normal (*n* = 55)	ASC-US (n = 3)	ASC-H (n = 4)	LSIL (n = 21)	HSIL (*n* = 10)	*p-*test for trends
HPV-16	6 (10.9)	1 (33.3)	1 (25.0)	5 (23.8)	5 (50.0)	< 0.001
HPV-18	1 (1.8)	0 (0.0)	1 (25.0)	0 (0.0)	0 (0.0)	0.30
HPV-31	4 (7.3)	0 (0.0)	1 (25.0)	4 (19.0)	2 (20.0)	< 0.001
HPV-45	4 (7.3)	0 (0.0)	1 (25.0)	4 (19.0)	3 (30.0)	< 0.001
HPV-51	6 (10.9)	1 (33.3)	0 (0.0)	2 (9.5)	0 (0.0)	0.17
HPV-52	13 (23.6)	0 (0.0)	1 (25.0)	3 (14.3)	3 (30.0)	0.04
HPV-33/58	9 (16.4)	2 (66.7)	1 (25.0)	4 (19.0)	3 (30.0)	< 0.001
HPV-35/39/68	17 (30.9)	1 (33.3)	0 (0.0)	5 (23.8)	3 (30.0)	0.05
HPV-56/59/66	18 (32.7)	0 (0.0)	0 (0.0)	11 (52.3)	6 (60.0)	0.05
HPV-16 only	4 (7.3)	0 (0.0)	1 (25.0)	0 (0.0)	2 (20.0)	0.44
HPV-18 only	0 (0.0)	0 (0.0)	0 (0.0)	0 (0.0)	0 (0.0)	0.30
HPV-16 or − 18	7 (12.7)	1 (33.3)	2 (50.0)	5 (23.8)	5 (50.0)	0.40
HPV- single infection	38 (69.1)	1 (33.3)	3 (75.0)	9 (42.9)	2 (20.0)	< 0.001
HPV- multiple infection	17 (30.9)	2 (66.7)	1 (25.0)	12 (57.1)	8 (80.0)	< 0.001
2 types	13 (23.6)	2 (66.7)	1 (25.0)	9 (42.9)	5 (50.0)	…
3 types	2 (3.6)	0 (0.0)	0 (0.0)	1 (4.8)	3 (30.0)	…
4 types	2 (3.6)	0 (0.0)	0 (0.0)	2 (9.5)	0 (0.0)	…

*p*-value < 0.05: statistically significant

Compared to those with single infections, women with multiple HR-HPV infections had a higher prevalence of LSIL (42.9% vs. 57.1%, *p* < 0.001) and HSIL (20.0% vs. 80.0%, p < 0.001). In women with multiple HR-HPV infections, the degree of severity of cytological lesions was strongly correlated with a decreased CD4 cell count (Additional file 1: Table S2).

**Fig. 3:**
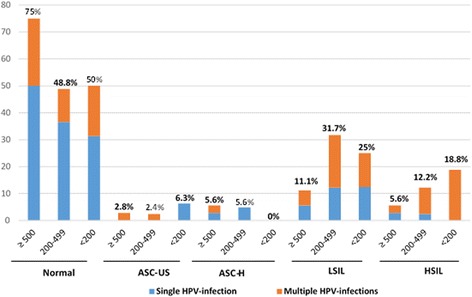
Prevalence of HPV infection according to CD4 counts and cytology and distribution of multiple HPV infections from 93 HIV-HPV positive women in Manaus, Amazonas

Figure 3 shows that in the HSIL category, women with CD4 counts ≥ 500 cell/mm^3^ had a lower proportion of multiple HR-HPV-infections (50.0%) than those with CD4 200–499 cell/mm^3^ (80.0%) and those with CD4 < 200 cell/mm^3^ (100.0%).

The overall agreement for conventional cytology observed between the blinded first and second evaluators was 89.0%. By type of lesion, it was > 90.0% and the PABA-kappa statistic ranged from 0.82–0.97 (Additional file 1: Table S3).

## Discussion

This study provides evidence about the prevalence of HR-PV infection, their associations, and cervical lesions among women living with HIV, an area of research with scarce literature from the Amazonas.

The prevalence of HR-HPV infection found, 31.1%, is approximately half of that found in another study in HIV positive women in Amazonas (61.6%) [18]. This discrepancy could be explained because in the latter study women were younger (median age: 32 years [IQR]: 27–38, vs. 40.7 years [IQR]: 33.1–46.2) and a peak among younger women has been described in a number of countries [19]. In the same study, women had a lower median CD4 cell count at enrolment (338.5 [IQR: 211.5–513.3] cells/mm^3^ vs. 504.0 [IQR: 321.0–676.5), which can limit the clearance of the virus, and 16.0% of women were commercial sex workers, which is a highly HPV exposed population [20]. Other studies in Brazil involving HIV positive women have found higher prevalence of HR-HPV from 35.7% to 98.0% [12, 21–24]. The differences with the results from other studies might be partly explained by information bias. Indeed, the study design used does not allow to differentiate between prevalent, incident and persistent HR-HPV infection leading to misclassification of the infection status and limiting comparability of our estimates with those from other studies.

We found that 43.0% of HR-HPV-infected women had multiple infections, which is in agreement with other Brazilian, ranging from 32.2% to 64.8% [24–27] and ranged from 52.0% to 64.3% in international studies [28, 29]. Women living with HIV are more likely to harbor multiple HPV infection than immunocompetent women [30] which is associated with an increased risk of intraepithelial neoplasia and of cancer [31, 32]. This probably reflects the effect of HIV-induced immunosuppression [33] rather than sexual risk behaviour [34].

In the present study, we found a wide diversity regarding HR-HPV types and their distribution, with HPV-52, HPV-16 and HPV-45 being the most frequent individual types. However, this needs to be taken with caution because the PCR method used did not allow the measurement for all individual genotypes and the various groupings of HPV types difficult the interpretation. As an example, the high prevalence of HPV-35/39/68 might be driven by HPV-35 alone and this type has been implicated in HSIL and invasive cervical cancer in women living with HIV [35, 36]. Women living with HIV are characterized by a wide variation of HPV genotypes, probably related to their sexual behaviour and the reactivation of latent infections, which can facilitate infection by different HPV genotypes. Contrary to what most studies suggest,[10, 12, 21, 28, 30, 37–39] HPV-16 was not the most common HPV type. This is in line with the results from other studies among indigenous populations in the Amazonas, [40] among women living with HIV in Brazil, [26] in the USA [29] and in Africa, [41] that corroborate our findings and place HPV-16 as not the most common type detected. It has been suggested that the contribution of HPV-16 correlates inversely with the overall HPV prevalence [42]. This pattern is explained by a higher prevalence of other HPV types in areas where HPV is extremely common, and the increase is not explained by the contribution of any other single type. Nevertheless, HPV-16 was the most prevalent individual HPV type among HSIL lesions, which is consistent with the results of a meta-analysis that included 19,883 women living with HIV from 86 studies worldwide [36]. This meta-analysis reported that HPV-16 positivity tended to increase with severity of cervical lesions. In Africa, HPV-16 accounts for 31.1% of HSIL and 46.6% of invasive cervical cancers. In Latin America, HPV-16 accounts for 37.5% of HSIL with no data available for invasive cervical cancers [36].

A low number of cases of HPV-18 were detected although it ranks amongst the top positions in most regions [37, 39]. In addition, HPV-18 accounts for a high proportion of HPV-positive in HSIL and invasive cervical cancers among women living with HIV [36]. Likewise, in other studies involving women living with HIV the HPV-18 contribution has been low [21, 24, 43–45]. It has either not been commonly detected in Brazilian studies [46, 47] or has shown a prevalence below 1.0% in asymptomatic women [48–50]. In two studies conducted in the Amazon region HPV-18 was not detected [37, 39]. It is unlikely that the low prevalence of HPV-18 found is related with the PCR method used. The BD Onclarity assay used showed good performance when compared with standard genotyping test [14, 15]. For HPV-18 (single or multiple infection), the agreement with the GP5+/6+ LMNX assay was high, with a kappa of 0.93 (95.0% CI: 0.87–0.99) [51].

Age-specific HR-HPV distribution presented as a unimodal distribution skewed to the left although this pattern was not statistically significant. Although there are studies that indicate a higher prevalence of HPV in younger women,[4, 52–55] we observed a higher prevalence of HR-HPV prevalence among older women (> 50 years) which has also been described in some other studies [10, 19, 32, 56–58]. It has been suggested that the increase in the perimenopause period may be due to higher rates of HPV persistence and recurrence at older ages rather than new HPV acquisition, [59] and that viral characteristics such as HPV type and variants, [60] weakened immune system, changes in sexual behaviour during middle age (both for men and women [61]), or previous individual screening practices, may play a role [56]. The association between younger age and higher prevalence of HR-HPV is probably due to the presence of transient infections in this group of women; however, this association is generally observed in non-HIV infected women but is not consistent in women living with HIV/AIDS [62].

In multivariable analysis, increasing age presented only borderline association with an increasing risk of presenting HR-HPV. We did not observe a peak of prevalence among young women (< 26 years) which might be explained because only 13.0% in our sample were < 26 years and among them, only 11 (27.0%) were infected with HR-HPV.

In the present study we found a clear association between weakened immune status and infection by HR-HPV. In most studies of HIV and HPV, the magnitude of increased HPV prevalence was proportional to the severity of immunosuppression [10, 18, 38, 63–65]. While many HPV infections are transient, women living with HIV are more likely to have persistent HPV infections, [66] and in other studies the frequency of persistence varied inversely with CD4 cell count [62, 65]. These results suggest that HIV induced immunosuppression might cytological findings was 21.9%, higher than the global estimate of 16.1% reported a meta-analysis for women in the general population from Latin America [42]. We found a high prevalence of LSIL (8.1%) and HSIL (3.4%) that reflects long-term persistent infections, in concordance with the high rates of multiple infections observed among HPV infected with these lesions [10, 25, 55]. Our results are consistent with other studies in which the presence of HPV in cytology with abnormal results ranged from 71.0% to 90.0% for LSIL [10, 67], and between 80.6% to 100.0% for HSIL [10, 30, 67]. Worsening of immune status was correlated with severity of lesions, as previously described [68].

We found high diversity of HR-HPV types in women with abnormal cytology results. In our study, most cytological alterations were related to types 16, 31 and 45. HPV-16 was the most common type in HSIL, which has been reported in a meta-analysis of HIV positive women with HSIL, [30] and in women from the general population [32]. Half of the HSIL cases presented HPV-16, which has high oncogenic potential and its presence is affected by the immunology status of the patient. The high prevalence of HR-HPV non-targeted by current vaccines does not reduce the importance of vaccination against HPV-16 and -18, proven genotypes with the highest carcinogenic potential. Furthermore, cross-protection has been described for HPV-45 and HPV-31 [69]. However, newer vaccines such as the nonavalent HPV vaccine present the possibility of better coverage for women [70–72].

This study has some limitations. It did not include a truly population-based design, as study participants were recruited from a reference hospital. However, this hospital attends to 95.0% of HIV patients from the Amazonas. Our sample included women who had a prolonged history of HIV infection (median 6 years), most were taking HAART (87.9%), had previously cytology (97.2%), had a relatively immune competence status (median CD4 cell count 504.0 cell/mm^3^), and median age was 40.7 years. These women could have a higher self-care standard, higher accessibility to health care and better health, which would result in underestimating the true HR-HPV prevalence in the population. In addition, we did not measure variables such as nutrition and behaviour of male partners that can influence HPV DNA detection [73–75]. The BD Onclarity ™ HPV Assay used for PCR does not allow a measurement for all individual genotypes. The various groupings of HPV types are difficult to interpret. It was not possible to investigate associations of duration of ART and the results of HR-HPV and cervical lesion, since the duration of ART would not be accurate given that treatment interruptions was not collected. Regarding the study size, the background estimate used was based on Brazilian studies [12, 22, 23] (ranging from 63.0% to 98.0%) but was higher than those reported in international studies. In addition, the statistical power was low at 80.0%. These reasons might have influenced the accuracy of the estimates measured and the strength of the association in the multivariable model. This study has a cross sectional design which allows only for presentation of baseline information.

## Conclusions

In conclusion, we found a high prevalence of HR-HPV infection and cervical lesions among women living with HIV in Amazonas. We found a wide diversity of HR-HPV genotypes, being the most common ones individually HPV-52, HPV-16 and HPV-45, although the highest prevalence was found in the genotype groups 56/59/66 and 35/39/68. HPV-16 and HPV-18 were less common than other HPV types but 50.0% of women with HSIL had HPV-16. The most important determinant of HPV infection was a low CD4 cell count. Most abnormal cytological findings were observed in women with poor immunological status. HPV quadrivalent vaccination used in Brazil might not offer protection for an important fraction of HPV-related disease burden in women living with HIV given the high prevalence of non-targeted vaccine HR-HPV, some of which (eg. 35, 39, 45, 56) contribute to high-grade lesions. Newer vaccines such as the nonavalent HPV vaccine [70–72] present the possibility of better coverage for women and will need to be evaluated. Strengthening preventive efforts is necessary to improve early detection through increasing accessibility to screening programs, adherence to follow-up among those with lesions, and intensifying health education for women living with HIV.

## Additional file

Additional file 1: **Table S1.** Prevalence of conventional cytology results according to HPV status and CD4 counts among 298 women living with HIV in Manaus, Amazonas. **Table S2** Association between cytological lesions and CD4 cell counts among women living with HIV in Manaus, Amazonas. **Table S3** Agreement between blinded observers in the independent reading of conventional cytology by type of lesion (DOCX 19 kb)

## Abbreviations

AOR: Adjusted Odds Ratio
ASC-H: Atypical Squamous Cells of Undetermined Significance, when it is not possible to disregard high degree lesions
ASC-US: Atypical Squamous Cells of Undetermined Significance
CD4: Molecule that is expressed on the surface of some T cells
CI: Confidence Interval
FCECON: Fundação Centro de Controle de Oncologia
HAART: Highly Active Antiretroviral Therapy
HIV: Human Immunodeficiency Virus
HPV: Human Papillomavirus
HR-HPV: High Risk HPV
HSIL: High-grade Squamous Intraepithelial Lesions
ICESP: Instituto de Câncer do Estado do São Paulo
LSIL: Low-grade Squamous Intraepithelial Lesions
OR: Odds Ratio
PCR: Polymerase Chain Reaction
SD: Standard Deviation
STI: Sexually Transmitted Infection
