# Supplementary material for: High risk human papillomavirus prevalence and genotype distribution among women infected with HIV in Manaus, Amazonas
Source: Virol J. 2018 Feb 17;15:36. doi: 10.1186/s12985-018-0942-6 (PMC5816532; doi:10.1186/s12985-018-0942-6)
Supplement: Supplementary file 1 — Table S1. Prevalence of conventional cytology results according to HPV status and CD4 counts among 298 women living with HIV in Manaus, Amazonas. Table S2 Association between cytological lesions and CD4 cell counts among women living with HIV in Manaus, Amazonas. Table S3 Agreement between blinded observers in the independent reading of conventional cytology by type of lesion (DOCX 19 kb) [file 12985_2018_942_MOESM1_ESM.docx]

**S1 Table. Prevalence of conventional cytology results according to HPV status and CD4 counts among 298 women living with HIV in Manaus, Amazonas**.

| Variables | Cytological results | | | | |
| --- | --- | --- | --- | --- | --- |
|  | **Normal**  **(n=251)** | **ASC-US (n=8)** | **ASC-H**  **(n=5)** | **LSIL (n=24)** | **HSIL (n=10)** |
| TOTAL (n=298) | 251 (84.2) | 8 (2.7) | 5 (1.7) | 24 (8.1) | 10 (3.4) |
| HPV -, CD4 cell count, cell/mm³ |  |  |  |  |  |
| <200 (n=11) | 9 (81.8) | 2 (18.2) | 0 (0.0) | 0 (0.0) | 0 (0.0) |
| 200-499 (n=79) | 74 (93.7) | 0 (0.0) | 0 (0.0) | 0 (0.0) | 0 (0.0) |
| ≥ 500 (n=114) | 112 (98.3) | 0 (0.0) | 0 (0.0) | 2 (1.8) | 0 (0.0) |
| Trend test | 0.01 | 0.001 | 0.40 | 0.64 | ... |
| Total (n=205) | 196 (95.6) | 5 (2.4) | 1 (0.5) | 3 (1.5) | 0 (0.0) |
| HPV +, CD4 cell count, cell/mm³ |  |  |  |  |  |
| <200 (n=16) | 8 (50.0) | 1 (6.3) | 0 (0.0) | 4 (25.0) | 3 (18.8) |
| 200-499 (n=41) | 20 (48.8) | 1 (2.4) | 2 (4.9) | 13 (31.7) | 5 (12.2) |
| ≥ 500 (n=36) | 27 (75.0) | 1 (2.8) | 2 (5.6) | 4 (11.1) | 2 (5.6) |
| Trend test | 0.04 | 0.60 | 0.42 | 0.12 | 0.14 |
| Total (n=93) | 55 (59.1) | 3 (3.2) | 4 (4.3) | 21 (22.6) | 10 (10.8) |

**S2 Table. Association between cytological lesions and CD4 cell counts among women living with HIV in Manaus, Amazonas.**

| **Cytological results** | | | | | | |
| --- | --- | --- | --- | --- | --- | --- |
|  | Normal | ASC-US | ASC-H | LSIL | HSIL | Test for trend |
| **Total (n=323)** |  |  |  |  |  |  |
| **N** | 274 | 9 | 5 | 25 | 10 |  |
| **CD4 cell count, cell/mm³** | 568 ± 306 | 316 ± 257 | 460 ± 284 | 367 ± 223 | 295 ± 226 | **0.001** |
| **HPV-negative (n=204)** |  |  |  |  |  |  |
| **N** | 195 | 5 | 1 | 3 | 0 |  |
| **CD4 cell count, cell/mm³** | 593 ± 309 | 262 ± 183 | 232 ± ... | 612 ± 252 | ... | **0.006** |
| **HPV-positive (n=93)** |  |  |  |  |  |  |
| **N** | 55 | 3 | 4 | 21 | 10 |  |
| **CD4 cell count, cell/mm³** | 524 ± 306 | 414 ± 419 | 517 ± 293 | 329 ± 206 | 295 ± 226 | **0.03** |
| **HPV- multiple infection(n=40)** |  |  |  |  |  |  |
| **N** | 17 | 2 | 1 | 12 | 8 |  |
| **CD4 cell count, cell/mm³** | 463 ± 253 | 619 ± 316 | 643 ± ... | 342 ± 234 | 238 ± 144 | **<0.001** |

**S3 Table. Agreement between blinded observers in the independent reading of conventional cytology by type of lesion.**

| Samples |  | + | - | Agreement | PABA Kappa | Positive  agreement | Negative  agreement |
| --- | --- | --- | --- | --- | --- | --- | --- |
|  | **n** | n (%) | n (%) | % |  | % | % |
| ASC-US | 324 |  |  | 90.9 | 0.82 | 25.6 | 95.2 |
| + | 20 | 5 (1.5) | 15 (4.6) |  |  |  |  |
| - | 304 | 14 (4.3) | 290 (89.5) |  |  |  |  |
| ASC-H | 324 |  |  | 98.7 | 0.97 | 0 | 99.4 |
| + | 3 | 0 (0) | 3 (0.9) |  |  |  |  |
| - | 321 | 1 (0.3) | 320 (98.8) |  |  |  |  |
| LSIL | 324 |  |  | 93.1 | 0.86 | 52.2 | 96.3 |
| + | 24 | 12 (3.7) | 12 (3.7) |  |  |  |  |
| - | 300 | 10 (3.1) | 290 (89.5) |  |  |  |  |
| HSIL | 324 |  |  | 96.2 | 0.92 | 33.3 | 98.1 |
| + | 8 | 3 (0.9) | 5 (1.5) |  |  |  |  |
| - | 316 | 7 (2.2) | 309 (95.4) |  |  |  |  |
